# Impact of new generation hormone-therapy on cognitive function in elderly patients treated for a metastatic prostate cancer: Cog-Pro trial protocol

**DOI:** 10.1186/s12885-017-3534-8

**Published:** 2017-08-16

**Authors:** Marie Lange, Heidi Laviec, Hélène Castel, Natacha Heutte, Alexandra Leconte, Isabelle Léger, Bénédicte Giffard, Aurélie Capel, Martine Dubois, Bénédicte Clarisse, Elodie Coquan, Frédéric Di Fiore, Sophie Gouérant, Philippe Bartélémy, Laure Pierard, Karim Fizazi, Florence Joly

**Affiliations:** 1 INSERM, U1086 ANTICIPE, Normandie University, UNICAEN, 14076 Caen, France; 2 Clinical Research Department, Centre François Baclesse, 14076 Caen, France; 3 Cancer and Cognition Platform, Ligue Nationale Contre le Cancer, 14076 Caen, France; 4 Medical Oncology Department, Centre François Baclesse, 14076 Caen, France; 5 Laboratory of Neuronal and Neuroendocrine Differentiation and Communication, Normandie University, UNIROUEN, INSERM, DC2N, 76000 Rouen, France; 6 UPO, Gustave Roussy, 94800 Villejuif, France; 7 NeuroHIV Rehabilitation Unit, Bicêtre University Hospital, 94275 Le Kremlin Bicêtre, France; 8 Normandie University, UNICAEN, EPHE Paris, INSERM, U1077, 14000 Caen, France; 9 Medical Oncology Department, Centre Henri-Becquerel, 76000 Rouen, France; 10 Digestive and Urology Oncology Unit, Rouen University Hospital, 76000 Rouen, France; 11 Medical Oncology and Hematology Department, Hôpitaux Universitaires de Strasbourg, 67000 Strasbourg, France; 12 Medical Oncology Department, Gustave Roussy, 94800 Villejuif, France; 13 Medical Oncology Department, CHU de Caen, 14000 Caen, France

**Keywords:** Cognitive impairments, Ageing, Prostate cancer, Hormone therapy, Adherence, Quality of life

## Abstract

**Background:**

New generation hormone-therapies (NGHT) targeting the androgen signalling pathway are nowadays proposed to elderly patients with metastatic castration-resistant prostate cancer (CRPCa). The impact of these treatments on cognitive function has never been evaluated whereas cognitive impairment may have an impact on the autonomy and the treatment adherence. The aim of this study is to prospectively assess the incidence of cognitive impairment in elderly men after treatment by NGHT for a metastatic CRPCa.

**Methods/design:**

The Cog-Pro study is a multicentre longitudinal study including CRPCa patients ≥70 years old treated with NGHT (*n* = 134), control metastatic prostate cancer patients without castration resistance treated with first generation androgen deprivation therapy (*n* = 55), and healthy participants (*n* = 33), matched on age and education. Cognitive, geriatric and quality of life assessment and biological tests will be performed at baseline, 3, 6 and 12 months after start of the treatment (inclusion time). The primary endpoint is the proportion of elderly patients receiving a NGHT who will experience a decline in cognitive performances within 3 months after study enrollment. Secondary endpoints concern: autonomy, quality of life, anxiety, depression, cognitive reserve, adherence to hormone-therapy, comparison of the cognitive impact of 2 different NGHT (abiraterone acetate and enzalutamide), impact of co-morbidities and biological assessments.

**Discussion:**

Evaluating, understanding and analyzing the incidence, severity of cognitive impairments and their impact on quality of life, autonomy and adherence in this group of patients with advanced disease is a challenge. This study should help to improve cancer care of elderly patients and secure the use of oral treatments as the risk of non-observance does exist. Our results will provide up-to date information for patients and caregivers on impact of these treatments on cognitive function in order to help the physicians in the choice of the treatment.

**Trial registration:**

NCT02907372, registered: July 26, 2016.

## Background

Prostate cancer (PCa) is a major public health issue. It is the second most common cancer and the second leading cause of cancer-related death among men worldwide [1]. PCa incidence increases with age, peaking at 70 to 74 years of age and PCa thus represents the most frequent cancer among male elderly patients. Although most PCa patients with metastatic disease initially respond to androgen deprivation therapy (ADT) leading to inhibition of gonadal testosterone biosynthesis, the pathology in a majority of patients progresses within the 2 years to a metastatic castration-resistant prostate cancer (CRPCa) [2].

Novel oral agents targeting the androgen signalling pathway (abiraterone acetate or enzalutamide) are available and are now proposed to patients with metastatic CRPCa in addition to first generation ADT. These new generation hormone-therapies (NGHT) were shown to improve 2 to 4 months the median overall survival rate of asymptomatic or pauci-symptomatic patients with a metastatic CRPCa [3]. According to the clinical trials, the main reported adverse events of these treatments were fatigue, hot flashes and oedema. Nevertheless, the impact of these treatments on cognitive function should be evaluated through a battery of cognitive tests, in order to establish a comprehensive knowledge of the impact of hormone-therapy in this cancer patient population particularly at risk.

Because of the better profile of tolerability compared to chemotherapy, new oral hormone treatments are largely prescribed to elderly cancer patients. Aging by itself is associated with some cognitive modifications, co-morbidities and functional decline which may have an impact on the autonomy and quality of life. Preliminary results suggest that elderly cancer patients would be at greater risk for increased age-related brain changes secondary to cancer and cancer treatments [4, 5]. There are still many unresolved questions, including the characterization of subgroups at risk to develop cognitive dysfunctions with cancer treatments, the impact of comorbidities, and the specific effects of different cancer treatments such as hormone-therapies. Even if there are some evidence of the impact of chemotherapy on cognitive function [6, 7] and more particularly in elderly patients and animal subjects [8, 9], the real impact of hormone-therapy on cognition is not well understood [10] and there are still important gaps in our knowledge and understanding of the mechanisms associated with cognitive disorders, especially in elderly cancer patients.

Prior studies described cognitive dysfunctions after ADT for PCa patients who performed significantly worse on visuomotor tasks than controls [11] and showed a cognitive decline mainly in visuospatial abilities and executive functions [10, 12]. After all, this between 47 and 69% of ADT-treated male patients who experienced some degree of impairment in at least one cognitive domain [10]. These findings are consistent with the known beneficial effects of testosterone on cognitive functioning in men [13]. For example, higher free testosterone levels have been found to be associated with better performance on objective neuropsychological tests of visuospatial processing, visual memory, visuomotor scanning, visuospatial abilities and episodic memory in healthy community-based samples of older men [14, 15].

Most of the cognitive studies conducted in PCa patients focused on non-metastatic situation, had small sample size and the impact of the age and co-morbidities has not been evaluated.

There are some argues to expect that NGHT prescribed in metastatic castrate resistance setting may induce cognitive dysfunctions among this particular vulnerable group of patients. Abiraterone acetate is a selective androgen biosynthesis inhibitor in the testis and the adrenal gland. In order to avoid cortisol deficiency, the treatment is combined with chronic use of prednisone. However, it has been well described that exogenous administration of corticosteroid may induce decline of cognitive performance via effects on medial temporal lobe and prefrontal brain areas [16]. In addition, the molecule may have an impact on the synthesis of dehydroepiandrosterone that is implicated in cognition in elderly men [13]. Enzalutamide binds and inhibits androgen receptors that are also present in the brain, including regions than can be severely affected such as the cerebral cortex and the hippocampus [17]. Some seizures have been already described in clinical studies with enzalutamide [18]. In rodents, it has been shown that androgens increase spine synapse density in the prefrontal cortex and the hippocampus [19, 20] and that disruption of the androgen receptors activity has an impact on aggressive behaviour [21]. Together, these observations suggest that androgen deprivation and/or androgen receptor inhibition would accelerate the aging process of PCa elderly patients.

These new hormonal agents with a better profile of toxicity than chemotherapy should constitute a better disease care option for older patients, as long as they are exhaustively evaluated and studied. Indeed, they are delivered on long lasting in daily oral route, thus the chronically developing troubles and the complete adherence of the treatments, especially in patients with cognitive impairment [22] must be investigated.

### Objectives

The purpose of the Cog-Pro study is to prospectively assess the incidence of cognitive impairment and cognitive complaints in elderly men (70 years old and over) after 3 months of treatment by NGHT for a metastatic CRPCa.

The secondary objectives are:

- To compare the impact on cognitive function of the two new available hormonal agents (abiraterone acetate and enzalutamide)
- To evaluate the impact of cognitive impairment on quality of life, autonomy and adherence to treatment,
- To evaluate the relationship between objective cognitive impairment and self-reported complaints of the patients, taking into account the cognitive reserve, anxiety, depression, and fatigue,
- To evaluate the relationship between co-morbidities, biological plasma markers and cognitive impairment.

## Methods/design

The Cog-Pro study is a multicentre longitudinal study.

Three groups of participants are enrolled: (1) patients of interest (metastatic CRPCa treated with NGHT) and two groups of controls, i.e. (2) control metastatic PCa without castration resistance patients (treated with first generation of ADT) and (3) healthy participants. The two groups of controls were matched (2: 1) to patients of interest on age and education. Inclusion and exclusion criteria are listed in Table 1.

**Table 1:** Study inclusion and exclusion criteria

	Patients of interest (metastatic CRPCa)	Control patients (metastatic PCa)	Healthy participants
Inclusion criteria	70-years old or more	70-years old or more	70-years old or more
	Patient must have a metastatic CRPCa	Patient with metastatic prostate cancer without resistance to castration	Man with no history of cancer
	Patient must have been already treated with first generation of ADT	Patient must have already started a first generation of ADT at least since 3 months	-
	Patient must be candidate for a treatment by a NGHT (with abiraterone acetate or enzalutamide), in combination with ADT	-	-
	Treatment with biphosphonates is authorized	-	-
	Patient must have not received chemotherapy except one line per Docetaxel for hormono-sensitive situation and which must be completed for a least 18 months before inclusion	Patient must have not received chemotherapy except one line per Docetaxel for hormono-sensitive situation and which must be completed for a least 18 months before inclusion	-
	OMS 0–2	OMS 0–2	Health status consistent with the participation to the study
	Patient must be asymptomatic or pauci-symptomatic (with pain control, Visual Analog Scale ≤3)	Patient must be asymptomatic or pauci-symptomatic (with pain control, Visual Analog Scale ≤3)	-
	No known brain metastasis	No known brain metastasis	-
	At least on level 3 (end of primary schools) of the Barbizet scale	At least on level 3 (end of primary schools) of the Barbizet scale	At least on level 3 (end of primary schools) of the Barbizet scale
Exclusion criteria	Neurological sequelae of traumatic brain injury, stroke, multiple sclerosis, epilepsy, neuro-degenerative disease…		
	Personality disorders and known progressive psychiatric disorder		
	Drug use and/or heavy drinking		
	Assessed to be unable or unwilling to comply with the requirements of the protocol		

*Abbreviations*: *CRPCa* castration-resistant prostate cancer, *ADT* androgen deprivation therapy, *NGHT* new generations of hormone-therapy

The patients of interest will be selected by physicians who will propose the study to the patients’ candidate for a NGHT according to the guidelines and the drug authorizations. The control group of patients will be selected by the medical oncologists and matched on age and education to the group of patients of interest.

The two groups of patients will be recruited in Cancer Comprehensive Centres for 2 years and during 1 year follow up. The healthy participants will be recruited from the general population through retirement associations, a senior university or an advertisement in Cancer Comprehensive Centres (husband of patients), for example. Participants enrolled in the study provide their written informed consent.

### Assessments

**Fig. 1:**
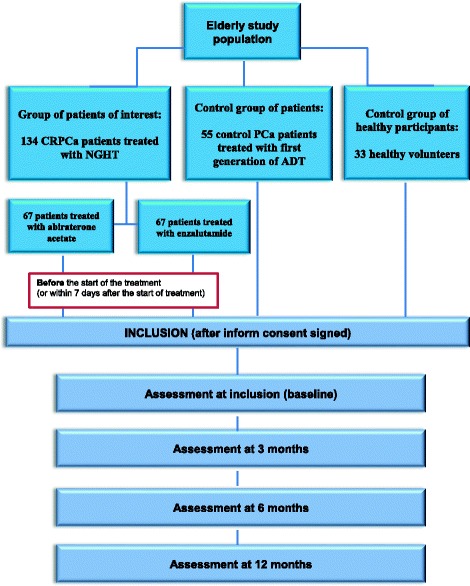
Study flowchart

For all the participants, assessments will be conducted, once signed the consent form, at inclusion - baseline - (for patients of interest: before the start of the treatment or within 7 days after the start of treatment), 3, 6 and 12 months (Fig. 1).

Cognitive tests, questionnaires and biological tests used for assessments are listed in Table 2.

**Table 2:** Used cognitive tests, questionnaires and biological tests

Evaluations	Before inclusion	At inclusion (baseline)^a^	3 months	6 months	12 months
Signed Informed Consent	✓				
Previous medical history	✓				
Cognitive assessment^b^					
MoCA					
Grober-Buschke test					
Digit span forward and backward (WAIS-III)		✓	✓	✓	✓
Code (WAIS III)					
Trail Making test					
Doors test					
Stroop Victoria					
Verbal fluencies					
Rey-Osterrieth Complex Figure					
Number location (VOSP)					
Years of education and fNART		✓ Only at inclusion			
Quality of life					
FACT-G, FACIT-F, FACT-Cog, HADS, ISI		✓	✓	✓	✓
Pain (VAS)	✓^a^	✓	✓	✓	✓
ONLY for PATIENTS (group of interest and control group)					
Geriatric assessment^c^					
G8					
Charlson					
ADL		✓	✓	✓	✓
IADL					
MNA					
Time up and go					
Quality of life					
FACT-P		✓	✓	✓	✓
Adherence evaluation^d^					
Morisky questionnaire			✓	✓	✓
Patient diary					
Biological tests^c^		✓	✓	✓	✓
Specific blood samples for further research^e^		✓			

*MoCA* Montreal Cognitive Assessment, *WAIS* Wechsler Adult Intelligence Scale, *VOSP* Visual Object and Space Perception Battery, *fNART* French National Adult Reading Test, *ISI* Insomnia Severity Index, *VAS* Visual Analog Scale, *ADL* Activities of Daily Living, *IADL* Instrumental Activities of Daily Living, *MNA* Mini-Nutritional Assessment

^a^For group of interest patients: before the start of the treatment or within 7 days after the start of treatment by abiraterone acetate or enzalutamide

^b^Cognitive assessment will be performed by neuropsychologists

^c^Geriatric assessment will be performed by a study nurse specialized in geriatric

^d^Had to be ≤ 3 on the 0–10 pain VAS scale to meet with inclusion pain criteria

^e^Adherence evaluation will be performed only in group of interest patients

^f^At each time: CBC, platelets, albumin, CRP, prealbumin, iron, ferritin, transferrin, creatinin, sodium, potassium, ALT, AST, GGT, ALP, total bilirubin, TSH, T4, testosterone. At inclusion only: cortisol (at 8 hours AM, fasting)

^g^1 EDTA (5 ml), 1 dry tube with gel (5 ml) and 1 dry tube without gel (5 ml)

At inclusion, previous medical history will be reported as well as relevant medications (psychotropic, opioids…).

#### Cognitive assessment

Objective cognitive function will be evaluated by the International Cognition and Cancer Taskforce (ICCTF) recommended battery of tests [7] and taking into account the previously observed cognitive impairment with ADT for PCa patients [10, 23]. The full evaluation will take less than one hour and will be performed by a neuropsychologist.

Global cognitive efficiency will be assessed by the Montreal Cognitive Assessment (MoCA: a rapid screening instrument for cognitive impairment) [24].

The main explored domains are visuo-spatial ability, episodic memory, working memory, executive functions and information processing speed (Rey-Osterrieth Complex Figure [25], number location (Visual Object and Space Perception battery - VOSP [26]), Grober-Buschke test [27], Doors test [28], digit span forward and backward (Wechsler Adult Intelligence Scale - WAIS III [29])), Trail Making test [30], verbal fluencies [31], Stroop Victoria [32], Code (WAIS III [29]). Proxies for cognitive reserve will include years of education and French National Adult Reading Test (fNART [33]).

#### Quality of life, adherence and pain assessment

We will use validated self-report questionnaires to evaluate global quality of life (Functional Assessment of Cancer Therapy-General: FACT-G [34]), fatigue (Functional Assessment of Chronic Illness Therapy-Fatigue: FACIT-F [35]), the prostate specific symptoms (Functional Assessment of Cancer Therapy-Prostate: FACT-P [36]), cognitive complaints (Functional Assessment of Cancer Therapy Cognitive Scale: FACT-Cog [37]), depression and anxiety (Hospital Anxiety and Depression Scale: HADS [38]) and quality of sleep (Insomnia Severity Index: ISI [39]).

We will combine subjective and objective measures to optimize the evaluation of adherence (assessed only in patients of interest). Adherence will be subjectively evaluated by a validated self-report questionnaire: the Morisky Medication Adherence Scale (MMAS) [40], 6-items French version [41] and objectively assessed by a patient diary.

Pain will be prospectively assessed with the Visual Analog Scale (VAS).

#### Geriatric assessment

Geriatric assessment will be performed by a study nurse in geriatric. The assessment consists of the G8 screening tool [42], the Charlson comorbidity index [43], the Activities of Daily Living (ADL [44]), the Instrumental Activities of Daily Living (IADL [45]), Mini-Nutritional Assessment (MNA [46]), Time up and go test [47].

#### Biological tests and biological collection

Biological tests will be performed only in the 2 groups of patients (Table 2). Specific blood samples (for plasma preparation) for the research will be collected at inclusion for constitution of a collection in patients who have giving their agreement on consent form (the specific blood sample is optional in the study).

#### Primary and secondary endpoints

The primary endpoint is the proportion of elderly patients receiving the NGHT who will experience a decline in cognitive performances (at least for one cognitive domain) within 3 months after inclusion.

The secondary endpoints will be based on:

- cognitive functioning (objective cognitive performances and self-report complaints),
- the comparison of the impact of the 2 new agents (abiraterone acetate and enzalutamide) on cognition,
- autonomy, quality of life, anxiety, depression and cognitive reserve,
- adherence to hormone-therapy,
- co-morbidities and biological assessments.

### Statistical analysis

#### Sample size

- Patients of interest (receiving NGHT)

Assuming between 47 and 69% of men who received ADT experience some degree of impairment in at least one cognitive domain [10], we hypothesise that 50% of patients will decline on cognitive performances.

To estimate the range of exact confidence interval (90%) with an accuracy of 7.5%, 121 assessable patients are required. To anticipate 10% of non-assessable patients, we plan to enrol 13 additional patients, for a total of 134 patients.

Given the secondary objective aiming at comparing the incidence of patients with cognitive decline between each available NGHT, study population will be constituted by:

- 50% of patients (i.e. 67 patients) receiving abiraterone acetate
- 50% of patients (i.e. 67 patients) receiving enzalutamide

With these sample sizes, it will be possible to detect (α = 5%; 1-β = 80%, bilateral test) some significant differences in proportions of patients with cognitive decline for the following proportions (π_1_, π_2_) or significant differences between patients and healthy participants.

- Control groups

In addition, two control groups matched on age and education with patients of interest will be included in order to have some neuropsychological data of reference:

- 55 control patients receiving ADT for metastatic PCa without castration resistance (Control patients),
- 33 healthy men (Healthy participants).

#### Planned analysis

Statistical tests and confidence intervals will be calculated with an overall error significance level of 5%. The proportion of elderly patients treated with NGHT who will experience a decline of cognitive functions (at least in one cognitive domain) between baseline and 3 months will be presented with its exact confidence interval.

The repeated measures analysis will be done using a mixed model.

Change in cognitive function will be determined, as recommended by ICCTF [7], using the standardized regression-based (SRB) approach used by Stewart et al. [48] and proposed by McSweeny et al. [49] and Sawrie et al. [50].

The secondary endpoints are based on quantitative scores of cognitive functions, quality of life, autonomy and geriatric frailty. Summary of score statistics and their significant variations from baseline will be calculated at each assessment time point for each group. The mean (and 95% confidence interval) and median (and min-max) of the absolute scores and changes from baseline will be reported.

Relationship between the objective cognitive impairment and following covariates:

1. autonomy, quality of life, anxiety, depression, fatigue, cognitive complaints, and cognitive reserve,
2. adherence to hormone-therapy,
3. two new agents (abiraterone acetate and enzalutamide),
4. co-morbidities and biological assessments,

will be assessed using a mixed model in order to compare change over time among the patient groups and healthy participants.

## Discussion

Due to the high incidence of prostate cancer, the increase of the life expectancy and the recent availability of NGHT for advanced prostate cancer, there is a dramatic increase of the number of elderly patients who are proposed to be treated for metastatic CRPCa. As these treatments improve the survival rate only of few months, it is very important to more extensively explore all their potential side-effects, especially in elderly patients. One major issue in this group of patients is to maintain autonomy and quality of life as long as possible, and this depends in a large part on their cognitive function at the baseline and during the period of cancer treatment.

We will conduct the first study evaluating cognitive function among elderly patients with metastatic CRPCa who will beneficiate from NGHT. By the end of the project, we should have assembled the complete information package on elderly patients at risk of cognitive decline with these new treatments. If we find that these treatments induce cognitive declines, it would be an important point to put into the balance of risk/efficacy of the treatments, and this point will have to be discussed with the patients. Also, it will provide opportunity to reinforce the follow-up of the adherence (i.e. follow-up by a clinician nurse for example). This research should therefore play a part in providing information as accurate as possible to the patients in term of quality of life, fatigue and emotional state during the treatment. Thus, a complete study to evaluate these cognitive impairments in elderly patients is necessary for maintaining a good quality of life in palliative situations.

This original study should help to better respond to some objectives of the third French cancer plan, such as improving cancer care of elderly patients and considering the specificities of this population, securing the use of oral treatments as the risk of non-observance does exist, taking into account and better understanding side effects of the treatments, such as memory and attention deficits.

## Conclusion

Evaluating, understanding and analyzing the incidence, severity of cognitive impairments and their impact on quality of life, autonomy and adherence among prostate cancer patients with advanced disease is a challenge. This study should help to improve cancer care of elderly patients and secure the use of oral treatments as the risk of non-observance does exist. Our results will provide up-to date information for patients and caregivers on impact of new generation hormone-therapies on cognitive function in order to help the physicians in the choice of the treatment and could promote the development of cognitively safe treatments.

## Abbreviations

ADT: Androgen Deprivation Therapy
CRPCa: Castration-Resistant Prostate Cancer
NGHT: New generation hormone-therapies
PCa: Prostate cancer
