# Correction to: Impact of new generation hormone-therapy on cognitive function in elderly patients treated for a metastatic prostate cancer: Cog-Pro trial protocol

**DOI:** 10.1186/s12885-017-3764-9

**Published:** 2018-01-30

**Authors:** Marie Lange, Heidi Laviec, Hélène Castel, Natacha Heutte, Alexandra Leconte, Isabelle Léger, Bénédicte Giffard, Aurélie Capel, Martine Dubois, Bénédicte Clarisse, Elodie Coquan, Frédéric Di Fiore, Sophie Gouérant, Philippe Bartélémy, Laure Pierard, Karim Fizazi, Florence Joly

**Affiliations:** 1 INSERM, U1086 ANTICIPE, Normandie University, UNICAEN, 14076 Caen, France; 2 Clinical Research Department, Centre François Baclesse, 14076 Caen, France; 3 Cancer and Cognition Platform, Ligue Nationale Contre le Cancer, 14076 Caen, France; 4 Medical Oncology Department, Centre François Baclesse, 14076 Caen, France; 5 Laboratory of Neuronal and Neuroendocrine Differentiation and Communication, Normandie University, UNIROUEN, INSERM, DC2N, 76000 Rouen, France; 6 UPO, Gustave Roussy, 94800 Villejuif, France; 7 NeuroHIV Rehabilitation Unit, Bicêtre University Hospital, 94275 Le Kremlin Bicêtre, France; 8 Normandie University, UNICAEN, EPHE Paris, INSERM, U1077, 14000 Caen, France; 9 Medical Oncology Department, Centre Henri-Becquerel, 76000 Rouen, France; 10 Digestive and Urology Oncology Unit, Rouen University Hospital, 76000 Rouen, France; 11 Medical Oncology and Hematology Department, Hôpitaux Universitaires de Strasbourg, 67000 Strasbourg, France; 12 Medical Oncology Department, Gustave Roussy, 94800 Villejuif, France; 13 Medical Oncology Department, CHU de Caen, 14000 Caen, France

## Correction

After publication of the original article [1] the authors found that Table 2 had been formatted incorrectly, meaning that some rows in the Table did not display the correct information.

An updated version of Table 2 is included with this Correction.

**Table 2:**
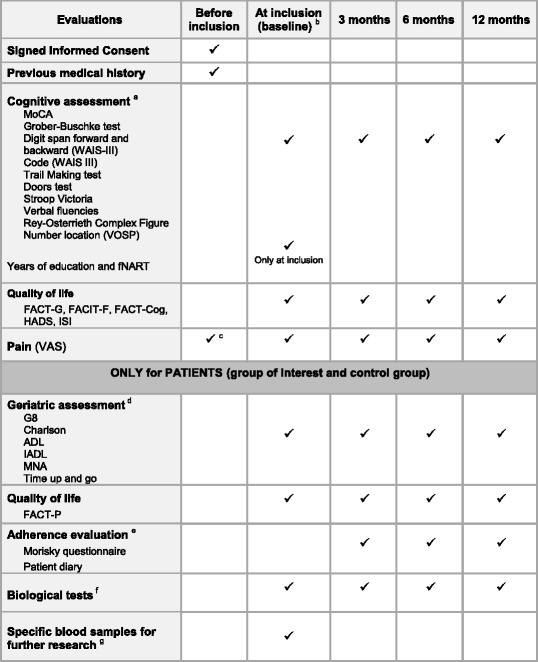
Used cognitive tests, questionnaires and biological tests

*MoCA* Montreal Cognitive Assessment, *WAIS* Wechsler Adult Intelligence Scale, *VOSP* Visual Object and Space Perception Battery, *fNART* French National Adult Reading Test, *ISI* Insomnia Severity Index, *VAS* Visual Analog Scale, *ADL* Activities of Daily Living, *IADL* Instrumental Activities of Daily Living, *MNA* Mini-Nutritional Assessment

^a^Cognitive assessment will be performed by neuropsychologists

^b^For group of interest patients: before the start of the treatment or within 15 days after the start of treatment by abiraterone acetate or enzalutamide

^c^Had to be ≤3 on the 0–10 pain VAS scale to meet with inclusion pain criteria

^d^Geriatric assessment will be performed by a study nurse specialized in geriatric

^e^Adherence evaluation will be performed only in group of interest patients

^f^At each time: CBC, platelets, albumin, CRP, prealbumin, iron, ferritin, transferrin, creatinin, sodium, potassium, ALT, AST, GGT, ALP, total bilirubin, TSH, T4, testosterone. At inclusion only: cortisol (at 8 h AM, fasting)

^g^1 EDTA (5 ml), 1 dry tube with gel (5 ml) and 1 dry tube without gel (5 ml)

The original article has also been updated.
